# Battle of Bull Run

**Published:** 1861-09

**Authors:** 


BATTLE OF BULL RUN—A VISIT TO THE WOUNDED.—This battle, fought on the 21st of July, will long be memorable in the history of human strife, both for its sanguinary character, and for having afforded the surgeons of this country the first great opportunity for studying the nature of gunshot wounds. Prior to that period, as stated in our July editorial, we really never had any severe engagements, either on land or on sea. The battles during the Revolution, and even those during the late war with Great Britain, resembled in their results, in a surgical point of view at least, skirmishes rather than great fights. The number of slain and wounded probably never, on any occasion, reached one-third the number of killed and wounded in July. The conflict at Bull Run must have been terrible; the slaughter immense. The forces on both sides are said to have fought with extraordinary bravery. The Minié ball, far more destructive than the round, and now almost exclusively used by the troops of all civilized nations, did immense execution. Hundreds upon hundreds must have been killed and wounded by shells, grape-shot, and cannon ball. Of sword and bayonet wounds the number seems to have been exceedingly small, as the men did not get into close quarters.

It is impossible, even now, nearly a month after the battle, to ascertain the number of wounded on either side. We have not seen even an attempt at the formation of a correct opinion upon the subject. During the hasty retreat of the Union army, nearly all those that were badly injured were left upon the field, and of course fell into the hands of the Confederates, where they still remain. Most of the rest found their way back to camp, or were picked up on the road, and sent to the hospitals at Alexandria, Washington, and Georgetown. During our visit to these institutions, forty-eight hours after the battle, we saw nearly four hundred of those unfortunate fellows. That this constituted only a small moiety of those who thus suffered may readily be imagined, if any confidence is to be placed in the report that many of our wounded are at Richmond and at Manassas.

The morning after our arrival at Washington, having learned that a large number of wounded were at Alexandria, we repaired at an early hour to that city, in company with Dr. Newell, Ex-Governor of New Jersey, and a highly intelligent member of the profession. Through the courtesy of Dr. A. Lawrence Sheldon, of the army, in charge of the hospital lately fitted up there by the government, we were permitted to accompany the assistants, Dr. McClellan and Dr. Thompson, into the wards, to examine the cases. The number of patients was one hundred and sixty-one, nearly all from Bull Run, the remainder having been wounded at the affair near Centreville, on the 18th of July.

Among the more interesting cases which we witnessed here, during the two mornings spent within its wards, were the following:—

1. William Grantman, aged 22, 1st Massachusetts Regiment, was shot on the 18th of July, in the engagement near Centreville, the ball, a round one, entering the back part of the left arm, about two inches and a half above the elbow, and ranging obliquely upward and outward, emerged a short distance from the axilla. It then passed into the chest immediately below the level of the axilla, and lodged behind, midway between the scapula and the spine, where it was cut out by Dr. Sheldon. The wound in the arm was subcutaneous. No bad symptoms supervened, and when we saw the man last, eight days after the receipt of the injury, he was in a fair way of recovery.

2. William Holland, aged 21, 2d Wisconsin Regiment, wounded at Bull Run, July 21st. The ball struck the jaw at its symphysis, breaking but not shattering the bone, knocking out two of the front teeth, and lodging on the right side, under the angle of the jaw, where it still was at the date of our last visit. Severe swelling, with fever, followed the injury; and, although the man was able to walk about the ward of the hospital, there was reason to believe that his convalescence would be tedious. The local symptoms were strongly denotive of the formation of an abscess in the upper part of the neck.

This man informed us that he had four brothers killed in battle, and that he was the son of a physician in Wisconsin; but, upon inquiry, it was found that he was remarkably illiterate, and even unable to write his name.

3. John Bedeman, aged 31, 1st New Jersey Regiment, wounded on the 18th of July. The ball, a large one, probably conical, passed through the upper part of the left thigh and the head of the penis, leaving four openings, those in the penis being separated merely by a small bridge of tissue. All the wounds were healing kindly, and the patient assured us that he had not suffered from any erections.

4. Vernon A. Marsh, aged 19, 2d Vermont Regiment, was shot on the 21st, the ball, a round one, striking the left side of the face, over the jaw, passing under the skin and muscles, and lodging on the opposite side, in the lower and back part of the neck, from which it was removed, soon after, by Dr. Palmer, the surgeon of the regiment. The jaw escaped fracture, and there was no bleeding in the mouth, indicative of injury of that cavity. With the exception of some pain on the left side, he was doing perfectly well, there being hardly any swelling along the track of the wound.

5. J. G. Cobb, aged 35, 71st New York Regiment, wounded on the 21st, probably by a shell, which carried away the left portion of the upper lip with a part of the corresponding side of the jaw and nose, causing a frightful gap, which, in the event of his recovery, must necessarily be followed by great deformity, remediable only by an extensive plastic operation. The nose, at the time of his admission, was hanging on the right side of the face. It was restored to its proper position, and secured by suture and adhesive strips. When we saw Cobb last, on the 26th of July, he was altogether comfortable, and in a fair way of rapid convalescence. His wound had bled considerably, and this was probably one reason why he was progressing so favorably.

6. Amos G. Scoffield, aged 28, 1st Minnesota Regiment; ball entered the back of the neck, on the left side, nearly midway between the mastoid process and spine, and a short distance below the level of the former, knocking out four of the upper teeth on the corresponding side, along with several pieces of the upper jaw. The wound bled considerably, but no bad symptoms followed.

At the *Georgetown Hospital*, under the charge of Dr. Gaenslen, of the army, and of his assistants, Dr. Frantz and Dr. Ingram, the following cases presented features of peculiar interest. At the time of our visit it contained one hundred and twelve patients laboring under gunshot wounds.

1. W. S. Rouse, aged 21, 2d Wisconsin Regiment, was wounded in the buttocks, the ball, a round one, weighing one ounce, entering on the left side, just behind the great trochanter, passing completely across, and then entering the opposite one, in the outside of which it was arrested just below the skin, from which it was removed by Dr. Frantz. The track made by the missile was long and deep, extending through the very thickness of the gluteal muscles. Of the three openings, one, namely, that of the first entrance, was round, the other two being somewhat slit-like. The patient had lost hardly any blood, and was rapidly recovering from the effects of his injury.

2. C. C. Dow, aged 25, 2d Wisconsin, shot through the neck on the right side, the ball entering just behind the sterno-mastoid muscle, two inches below the mastoid process, and emerging at the mouth, injuring, in its passage, the tongue on the right side, and knocking out the left inferior lateral incisors and also the corresponding teeth of the upper jaw. The parts bled considerably for some time after the accident, but, with this exception, and some difficulty of deglutition, no other inconvenience was experienced.

3. George Bugby, aged 25, 15th Connecticut Regiment, shot on the 16th of June, at Vienna, the ball entering the shoulder-joint in front and emerging behind, cutting off the head of the humerus at the anatomical neck, and shattering the bone for an inch lower down. Resection was performed three weeks after the receipt of the injury, two inches and a half of the bone being removed. The glenoid cavity was found to be sound. The man seemed to be doing well for a week, when secondary hemorrhage took place, and, recurring several times, caused excessive prostration, with a probability of a fatal result.

Since writing the above, Dr. Frantz, in a letter, dated August 17th, has informed us that Bugby has not had any further hemorrhage, and that he is regarded as out of danger.

4. Stephen P. Russell, aged 23, 2d Michigan Regiment, accidently wounded on the 15th of July, by a pistol shot, the ball passing in at the lower part of the sternum, just above the ensiform cartilage, a little to the left of the middle line. He was sitting, when shot, against a tree, the man who wounded him standing up. Very little bleeding followed the injury, but severe pain was at once felt, radiating toward the left shoulder and through the abdomen, and for the first four days there was difficulty in voiding the urine. Pretty active peritonitis supervened, lasting for the better part of a week, and thus rendering it extremely probable that the ball, apparently a small round one, had passed somewhere into the abdomen, although its precise route and destination could not be ascertained. The man on the 26th of July was rapidly convalescing.

5. Willliam Judkins, aged 38, 3d Maine Regiment, was injured in the left thigh by the explosion of his musket, inflicting a frightful muscular wound at least ten inches in length by four in width. The accident happened on the 21st of July, and when we saw him on the 26th, the parts had a dark, unhealthy appearance, with marks of erysipelas in the skin, but no signs of suppuration. There was no excitement in the pulse, but the man looked badly and was occasionally flighty. He expired two days after our visit.

6. James McIntyre, aged 35, 2d Wisconsin Regiment, was shot, on the 21st July, in the left arm, near the centre of the deltoid muscle, the limb being raised at the time. The ball, a large conical one, passed through the lungs, and lodged behind, on the right side, below the level of the scapula, and a short distance from the spine, making a track at least eighteen inches in length. It was cut out on the same day. The man immediately spat and coughed up blood, and continued to do so for some time after the receipt of the injury. The left lung was invaded with emphysema, the respiration was hurried and painful, and there was dullness on percussion of the chest.

In a short time the emphysema extended over all the left side and back, and the man became feverish. Notwithstanding this, however, he walked a distance of twenty-two miles after he was shot. When we saw him last, five days after the accident, he was in an excellent condition, and in a fair way of recovery.

7. Jesse W. Burgess, aged 25, 1st Connecticut Regiment, was struck on the left forearm with a conical ball which, passing obliquely in front of the elbow, without opening the joint, emerged a short distance above, and then penetrated his vest, burying itself in his watch, a strong silver one, the case of which was completely indented by it. The ball itself was much flattened and very irregular. Except knocking his breath out of him, as he expressed it, and causing a good deal of pain in the side for a few days, Burgess experienced no particular inconvenience from his mishap. The skin was hardly visibly bruised. He left Georgetown on the 29th of July, calling, at the kind request of Dr. Frantz, at our office the next morning on his way home.

At the *Seminary Hospital*, Georgetown, under the supervision of Dr. Joseph K. Smith, of the army, the only case, out of upwards of seventy, of special interest to us, was that of Thomas Thompson, aged 22, of the New York Regiment of Fire Zouaves. His forearm had been struck by a round ball, which entered in front of the limb, about four inches above the wrist, and, passing round the radius, lodged under the skin of the dorsum of the hand, without fracturing any of the bones.

At Washington City we visited the three hospitals opened there for the accommodation and treatment of the wounded. At the *Columbian Hospital*, under the charge of Dr. Abadee, and his assistants, Drs. Asch, Brainard, and Knickerbocker, the number of surgical cases from the battle-field was under twenty, and, excepting a single one, without any special interest. The case to which we allude was that of a young man who had been struck on the abdomen, nearly midway between the umbilicus and pubic symphysis, by a small ball which buried itself internally, but no one could tell precisely where. From the fact, however, that there was for a number of days a considerable degree of tenderness in the left iliac region, with some evidences of peritonitis, the supposition was that the missile had lodged in the iliac bone. The man was doing well when we saw him some days after the accident. He had not had any difficulty in his bladder.

At the *C Street Hospital* the number of wounded from gunshot was about forty. Through the kindness of Dr. Getty, of the army, and of his assistants, two cases of great interest were pointed out to us here.

1. Sergeant Johnson, aged 30, of Rickett's Battery, was struck *en ricochet* on the right arm by a twelve-pound shot which broke the humerus at three different places, within a few inches of each other, without lacerating the skin, or even causing any considerable ecchymosis. He was doing well.

2. Philip Kernan, aged 25, 5th Artillery, was accidently shot on the 26th of July, with a horse-pistol, in the back, the ball entering on the right side, near the posterior border of the scapula, passing across the superior part of the chest, and emerging on the left side of the neck, just below the cricoid cartilage, laying open the trachea, as was shown by the fact that air issued at both orifices. The left side of the chest and trunk was emphysematous, and there was occasionally a little cough of bloody froth and mucus. No hemorrhage occurred, but the shock was very severe, although the reaction was complete in an hour and a half after the receipt of the injury. Twenty-four hours after the accident all the symptoms were favorable.

At the *Washington Infirmary*, formerly under the control of the medical faculty of Washington College, but now in charge of Dr. White, of the army, and of his assistants, Dr. Butler and Dr. Gouley, upwards of fifty cases of gunshot wounds had been admitted at the time of our visit. Two cases in particular attracted our attention here, and are worthy of being noticed.

1. A young German, about 22 years of age, had received a wound in the shoulder-joint, the ball passing, if we mistake not, obliquely from before backward, laying open the articulation, and inflicting serious mischief upon the head and neck of the humerus. Two days after the accident an exploratory incision was made, with a view of ascertaining the extent of the injury, and the necessity or non-necessity of resection. The mischief appearing comparatively slight, the operator contented himself with the removal of a few small splinters of bone, trusting that, as the patient was a stout, robust, temperate man, he would be able to surmount all obstacles to a cure. In a short time, however, the parts began to swell enormously, and upon cutting again into them it was found that the injury had been far more extensive than had at first been supposed. Amputation was accordingly performed at the shoulder, with what result we do not know.

2. The other case in this hospital that interested us very much was that of Colonel Farnham, of the New York Zouaves, who was struck on the head at Bull Run, on the 21st July, the ball glancing and slightly bruising the scalp. We saw him on the 25th; he was lying upon his bed, holding a book in his hand, and interested in what he was reading. He was disposed to converse, was free from pain and fever, and was not supposed by any one to be in danger. Subsequently to this, however, he was seized with head symptoms; he became excessively restless, complained of severe cephalalgia, and was finally seized with delirium. After lingering in this way, he expired on the 14th of August, a little upwards of three weeks after the receipt of his apparently trifling injury.

No one in visiting these institutions could fail to be struck with the monotonous nature of their cases of gunshot injuries. With few exceptions, the wounds were situated in the extremities, and, apparently, with nearly equal frequency in the upper and lower. One man, for example, was shot in the finger, thumb, or hand; another in the forearm, wrist, or elbow; a third in the arm or shoulder; and so in regard to the foot, leg, and thigh. Of the numerous cases, amounting altogether to nearly four hundred, not a solitary one was shot in the head and spine; only two in the chest and abdomen, and none in the pelvis. No large vessels seemed to have been laid open in any; very few had broken bones; two had been shot through the shoulder-joint. From all this it is evident that those most severely wounded had been left upon the field of battle. From a careful examination of the cases our conviction was that nearly all — certainly ninety-five per cent. — would recover. During our last visit to them they were, with five or six exceptions, in the best possible condition for a speedy convalescence. Erysipelas, it is true, was just beginning to make its appearance in some of the wards, both at Alexandria, Washington, and Georgetown, but did not spread to any extent or exhibit any virulence.

The treatment in all the hospitals was a pretty full diet with rest and occasionally a mild purgative, and cold water as the local application. So universally was this remedy employed, and such were its happy effects, that it would have done the spirit of old Biondo good to witness it, to say nothing of Kern, Larrey, Hennen, Guthrie, and other great military surgeons. Under this treatment very few of the patients had any fever, and very few of the wounds any disposition to suppurate. Indeed, in many there was no discharge of pus at all.

We felt much interested in inquiring into the amount of pain experienced by some of the patients at the moment they were shot, and were not surprised at their answers. Most of them stated that the passage of the ball produced merely a slight burning or smarting sensation; some few felt pretty severe pain, while others did not suffer any at all. In some cases there was a consciousness, at the moment, of a certain degree of numbness or weight in the part or for some distance around and beyond.

The hemorrhage was for the most part very slight, and but few of the cases suffered much from shock. What was remarkable, many of the men, even of those who were pretty severely wounded in the thigh and leg, walked —shall I say ran ?—all the way from Bull Run to Alexandria and Washington. We have already given the particulars of the case of a man shot through the lungs who walked twenty miles after he was wounded, and that apparently without being the worse for it.

With regard to the openings of entrance and exit, the differences were usually well marked and unmistakable. There was only now and then an instance of doubt or uncertainty, requiring a careful examination in order to determine the point. In general, the orifice of entrance was round and well defined, with or without slight inversion of its edges, and occasionally a little discoloration of the neighboring surface from the effects of the powder on the ball. The opening of exit, on the contrary, presented more the characteristics of a slit or rent, with a certain degree of eversion or prominence, and without any blacking of the skin. These phenomena were present, as a general rule, whether the wound was caused by the round or the conical ball. The orifice of egress, however, was usually disproportionably greater, as well as more irregular, in the latter case than in the former. When the wound was subcutaneous, or chiefly so, we noticed that this opening was sometimes of enormous dimensions, and at the same time remarkably ragged, as if the parts had been roughly lacerated.

During the healing process we noticed that the parts around the orifice of exit were generally less inflamed than around the orifice of entrance, which were occasionally in a slightly sloughing condition. In none of the cases, however, that fell under our observation was there anything like extensive mortification.

It is well known that terrible and even fatal injury is not unfrequently inflicted by partially spent cannon shot coming in contact with the surface, extensively lacerating the soft parts and comminuting the bones, if not actually pulpefying these structures, and yet not perhaps even bruising the skin, much less tearing it. These effects were formerly ascribed to what was called the windage of a ball, or the forcible concussion of the air. A case bearing upon this point occurred at the battle of Bull Run, in a man of the name of Cornelius Laroe, aged 41, of the 2d Wisconsin Regiment, an inmate of the hospital at Alexandria. His cap was knocked off by the ball, which he heard whistling as it passed his ear. He was instantly seized with deafness on the corresponding side, along with a sense of weight in the head. The shock strongly convulsed his whole frame, but, regaining his equilibrium, he continued at his post until after the engagement, suffering, however, very severely with pain. When he was admitted, the cheek was swollen and slightly discolored, and so much inflammation afterward arose as to make it difficult to prevent gangrene.

In concluding this brief notice of a visit which, to us, was full of instruction, we tender our very cordial acknowledgments to the chiefs and their assistants of the different hospitals alluded to, for the courtesy they extended to us in promoting the object of our inquiries. Our solicitude to see the sufferers at Bull Run, and to examine their cases while their wounds were still fresh, was very great—not as a matter of idle curiosity but of scientific study—and we assure these gentlemen we shall never forget their kindness. We were greatly surprised not to see any more interest manifested in the subject by other surgeons. With the exception of Professor Blackman, of Cincinnati, and Dr. Newell, of New Jersey, we observed not a single prominent member of the profession during our hospital tour.

